# Cystic appearance - a new feature of solid fibrous tumours in the lacrimal gland: a case report with literature review

**DOI:** 10.1186/s13000-019-0845-x

**Published:** 2019-06-22

**Authors:** Ancuta-Augustina Gheorghisan-Galateanu, Dana Cristina Terzea, Iulia Burcea, Roxana Dusceac, Cristina Capatina, Catalina Poiana

**Affiliations:** 1 Department of Cellular and Molecular Biology and Histology, “Carol Davila” University of Medicine and Pharmacy, 050474 Bucharest, Romania; 2 Department of Pathology, “C.I.Parhon” National Institute of Endocrinology, 011863 Bucharest, Romania; 3 Department of Endocrinology, “Carol Davila” University of Medicine and Pharmacy, 050474 Bucharest, Romania

**Keywords:** Cystic changes, Solitary fibrous tumour, Lacrimal gland, Exophthalmia

## Abstract

**Background:**

Solitary fibrous tumours (SFTs) rarely occur in the orbit, especially in the lacrimal area. These tumours are mostly solid. Cystic changes have been documented, but they remain very rare. Only three cases of primary orbital solitary fibrous tumours with cystic changes have been reported in the literature, but no cases have been reported to occur in the lacrimal gland. Solitary fibrous tumours generally follow a benign course and are treated definitively with surgical excision. Data from the literature suggest that the cystic nature of SFT presents a risk of recurrence and could be a harbinger of malignancy.

**Case presentation:**

A 42-year-old woman was admitted to the endocrinology department for right unilateral exophthalmia and epiphora in the last 8 months. An ophthalmological evaluation showed exophthalmia only in the right eye (22 mm) and normal visual acuity, visual field and extraocular movements. Investigations revealed normal thyroid function. Orbital magnetic resonance imaging detected a 4 × 2,2 × 2,7 cm septate pseudocystic mass in the right lacrimal gland. Given her lacrimal gland tumour diagnosis, the patient was submitted for neurosurgical intervention with total ablation of the tumoural mass and complete right dacryoadenectomy. Although the intraoperative extemporaneous examination results were suggestive of a haemangiopericytoma, histological and immunocytochemical examination showed an extrapleural SFT. The postoperative clinical evolution was favourable, with remission of the exophthalmia. Fifteen months after surgery, no signs of recurrence were noticed.

**Conclusions:**

We report the first case of an SFT with cystic changes in the lacrimal gland. Although the presence of cavitary lesions alone does not necessarily indicate aggressive behaviour, cystic changes pose a risk of recurrence and may suggest malignant transformation over time. As a result, our case requires long-term follow-up due to recurrence and malignant potential.

## Introduction

Although until a few years ago the most frequent site of solitary fibrous tumours (SFTs) was thought to be the intrathoracic region, with a peculiar predilection for body cavity sites, this tumour can develop in any part of the body where mesenchymal cells are present. Orbital involvement was first independently reported in 1994 by Dorfman et al. and Westra et al., respectively [1, 2]. Until now, 90 cases of orbital SFTs have been reported in ophthalmologic and pathologic journals [3]. SFTs can be found anywhere in the orbit, but they have rarely been observed and described in the lacrimal gland. Since 1996, when Scott et al. reported the first SFT in the lacrimal gland fossa, to our knowledge, only 15 such cases of SFT in the lacrimal gland and lacrimal gland fossa have been reported, with half of them reported in the last five years (Web of Science, Pub Med and Google Scholar). All SFTs in the lacrimal area have been communicated as isolated case reports. Orbital SFTs are mostly solid, and cystic tumours are very rarely observed. To date, no case of a cystic SFT in the lacrimal gland has been described. This new feature of the tumour requires closer monitoring of the patient because of the risk of recurrence and malignant transformation.

## Materials and methods

We introduce a case of lacrimal SFT with cystic changes and a brief literature review regarding this specific topic. This research is based upon the collection of data from all cases of SFT involving the lacrimal gland area reported in the English-written literature starting with the first case reported in 1996 and including our current case.

## Results

### Case presentation

**Fig. 1:**
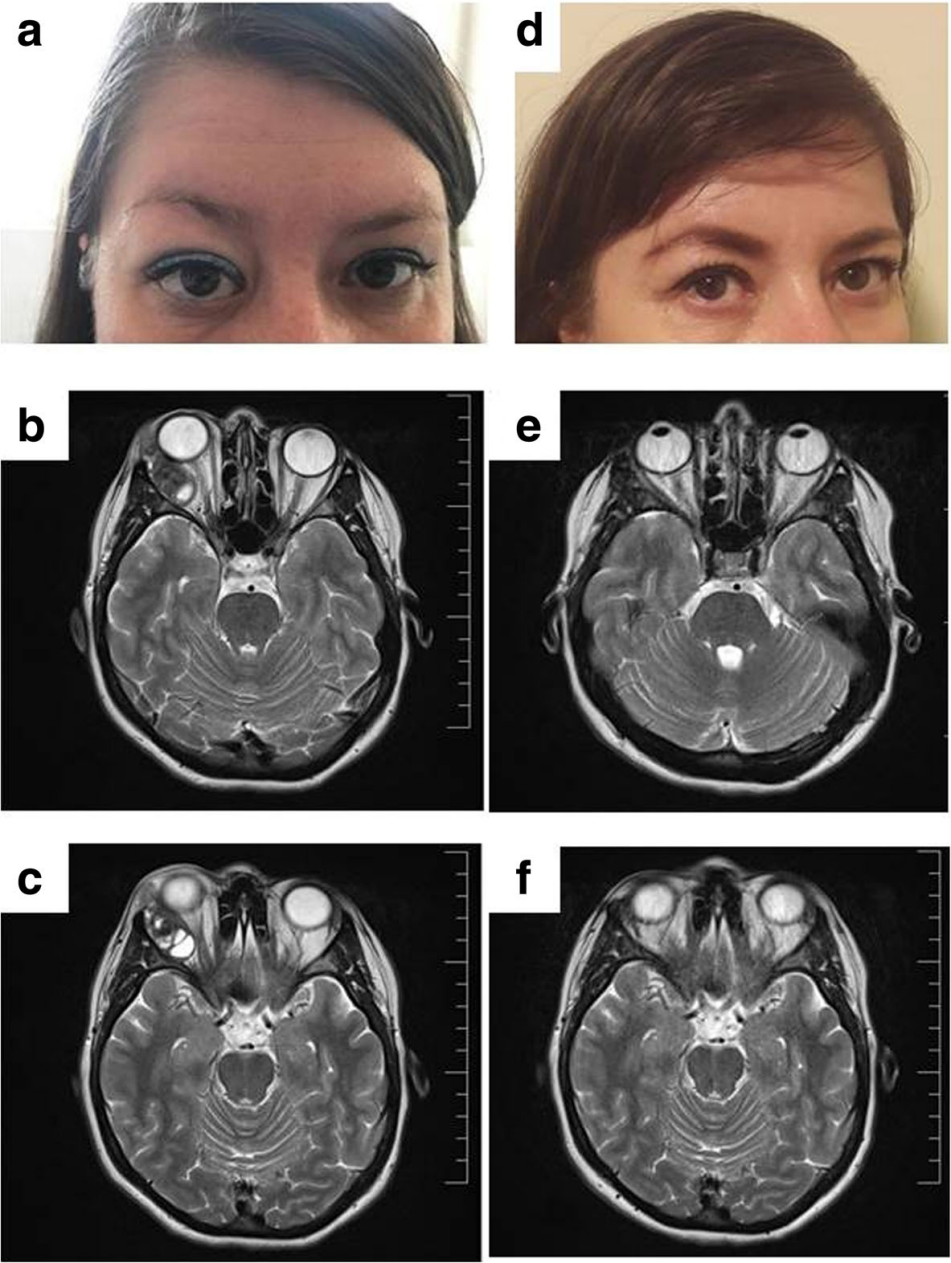
**a** Preoperative photograph of the patient showing right exophthalmia. **b, c** MRI detected a well-defined 4 × 2,2 × 2,7 cm mass in the right lacrimal gland region that showed a septate pseudocystic appearance and exerted a mass effect on adjacent structures without local bony destruction. **d** Postoperative case photograph showing normalization of the globe and resolution of right exophthalmia. **e, f** No evidence of residual tumour was found in the orbital MRI control

We present the case of a 42-year-old Caucasian non-smoking female who was admitted in the Endocrinology Department for right unilateral exophthalmia and epiphora in the prior 8 months (Fig. 1a). Her family clinical history was positive for breast cancer (mother) and high blood pressure and type 2 diabetes (father) but negative for endocrine conditions. Since menarche at age 12, she has had regular menses and a childbirth.

### Medical records

In adolescence, the patient was diagnosed with allergic rhinitis, for which she received inconstant Xyzal. There was no inflammatory disease or previous trauma in the anamnesis.

### On admission

On physical examination, the patient’s body mass index (BMI) was 24,8 kg/m^2^ and her clinical data were normal. The ophthalmological evaluation showed only exophthalmia of the right eye (22 mm) and normal visual acuity, visual field and extraocular movements. No optic nerve atrophy or papilledema was found. The neurological exam was normal. No abnormal findings were identified on current laboratory tests, including a full blood count, erythrocyte sedimentation rate, glycaemia, renal profile, and liver function. The hormonal thyroid profile was within the normal range and showed no autoimmune abnormality. Ultrasonography of the anterior cervical region showed a thyroid with normal volume and structure. Under these circumstances, an orbital magnetic resonance was recommended. A well-defined 4 × 2,2 × 2,7 cm mass in the right lacrimal gland with septate pseudocystic appearance, small haemorrhagic areas, heterogeneous structure, and mass effect on adjacent structures without local bony remodelling was detected (Fig. 1b, c). The preoperative diagnosis was a lacrimal gland tumour.

### Surgical approach

**Fig. 2:**
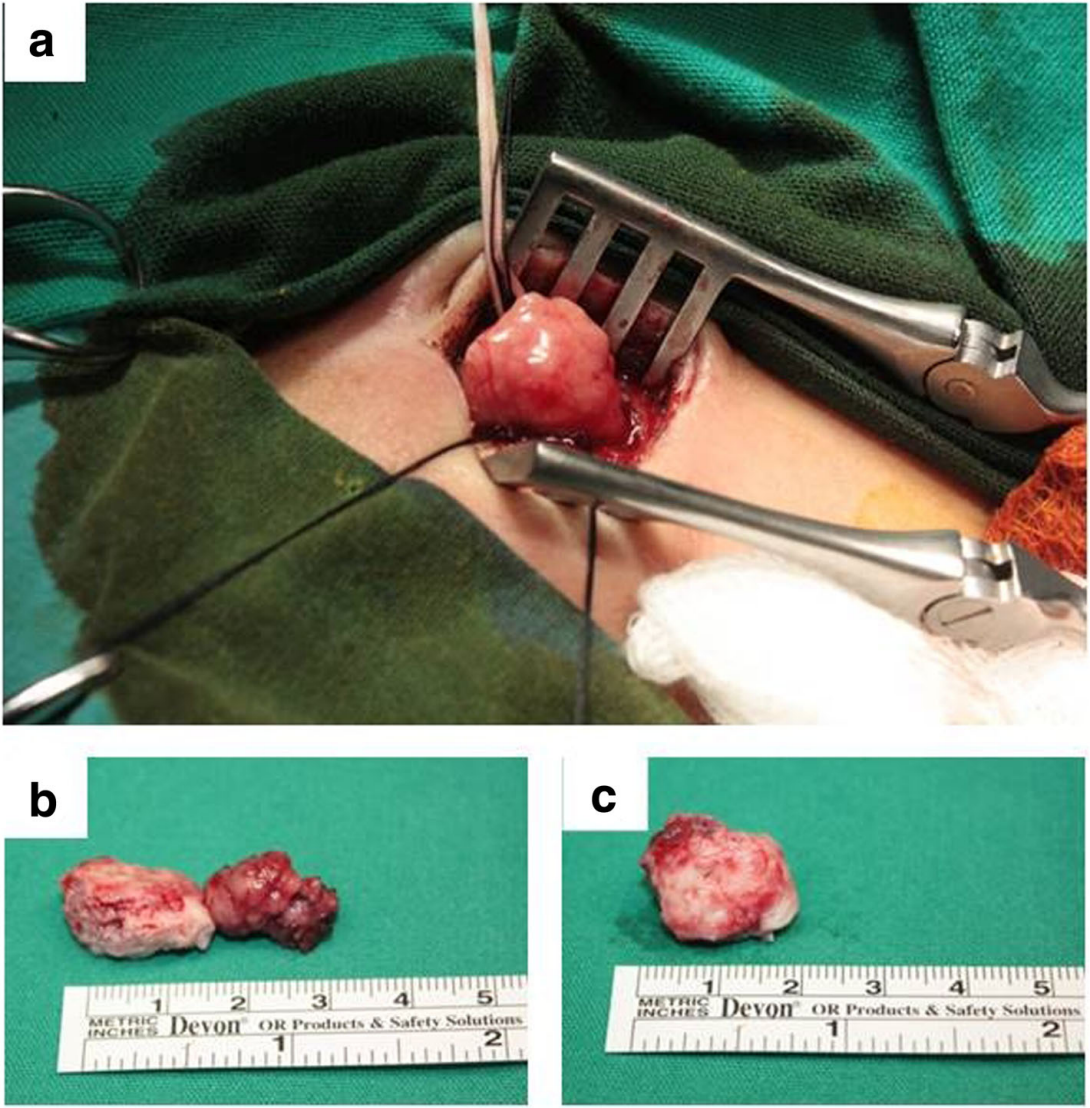
**a** Intraoperative image: the tumour was discovered in the right lacrimal fossa. **b** Gross photograph of the tumour and right lacrimal gland following excision. **c** Macroscopic aspect of the excised tumour mass showing its bosselated surface, purple greyish colour with yellow areas and elastic consistency. It was 2,5 × 1,7 × 1,5 cm in size

Based on this diagnosis, the patient was referred to a neurosurgical department. The lesion was approached extraperiosteally through a sub-brow incision. The tumoural mass with swollen lacrimal gland was identified in a clear dissecting plane and isolated from the surrounding muscles and showed no local infiltration (Fig. 2a). Total ablation of the tumour and complete right dacryoadenectomy was performed (Fig. 2b).

### Pathological report

**Fig. 3:**
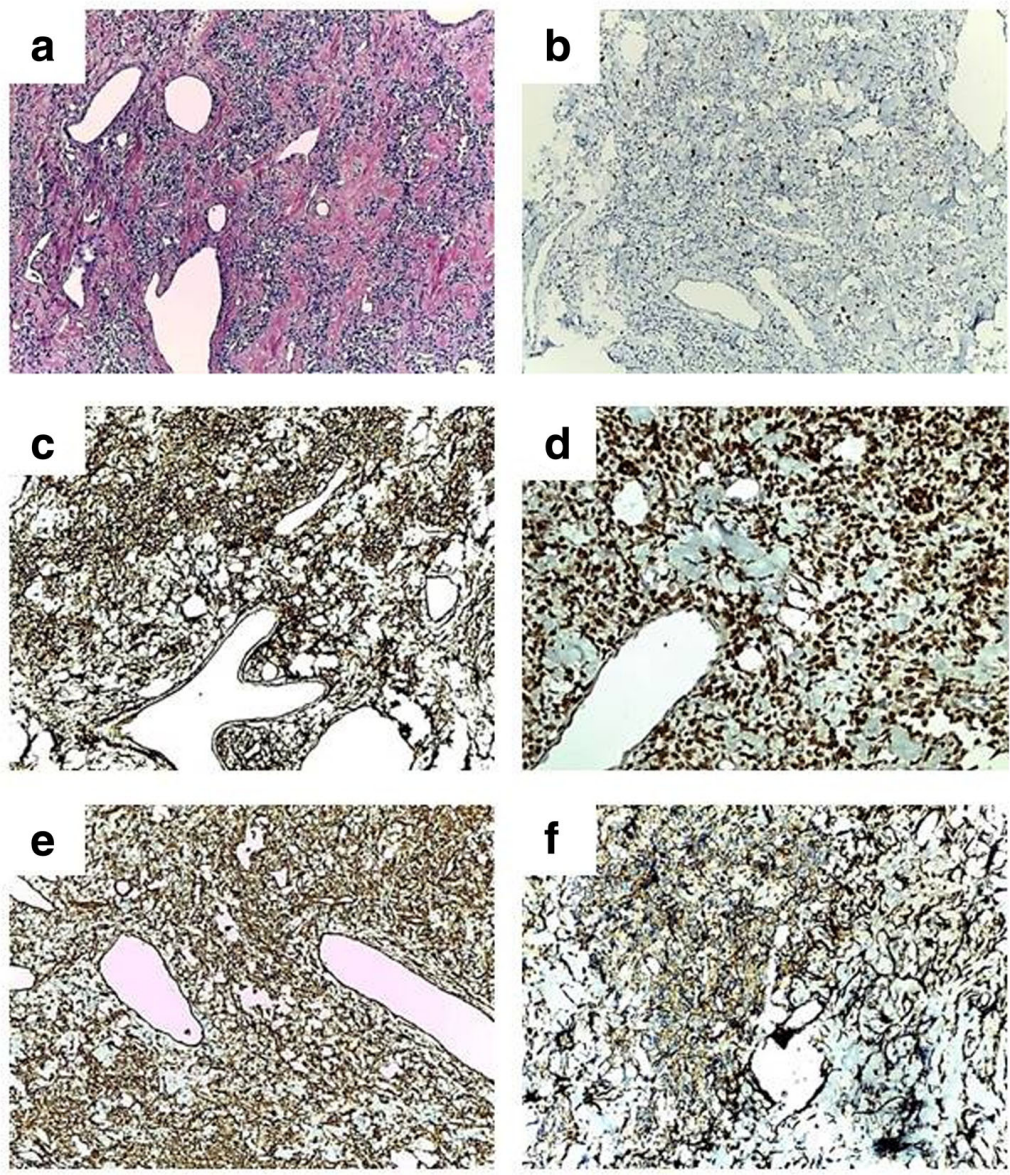
**a** Microscopic examination showed areas with hyper- and hypocellularity with large areas of collagen and frequent blood vessels, some of which had thin walls, a branched appearance and dilated lumens (haemangiopericytoma-like pattern) (HE stain × 100). Immunohistochemical staining showing weak positivity for Ki67 (× 100) (**b**), strong and diffuse positivity for CD34 (× 100) (**c**) and STAT6 (× 200) (**d**), and focal positivity for vimentin (× 100) (**e**) and CD99 (**f**) (× 200)

Macroscopically, the tumour specimen was well-circumscribed and had a bosselated surface, a purple greyish colour, yellow areas and an elastic consistency and measured 2,5 × 1,7 × 1,5 cm in size (Fig. 2c). Intraoperative extemporaneous examination was suggestive of a haemangiopericytoma. Paraffin tissue blocks were prepared after fixation and routine processing steps. Hematoxylin-eosin staining was performed. Histopathological examination showed cellular proliferation of variable density and spindle or oval-shaped cells with eosinophilic cytoplasm that was sometimes vacuolated. The nuclei were oval-shaped with small visible nucleoli. The mitotic activity was reduced. The stroma contained large areas of collagen and frequent blood vessels of variable calibre; some of these had thin walls and a branched appearance (Fig. 3a). The diagnosis was highly suggestive of SFTs arising in the lacrimal gland. Immunohistochemical staining showed a Ki67 index of 8% in the tumour cells (Fig. 3b), strong and diffuse positivity for CD34 (Fig. 3c) and STAT6 (Fig. 3d), and zonal positivity for vimentin (Fig. 3d) and CD99 (Fig. 3f). Epithelial membrane antigen (EMA) was positively expressed in rare, dispersed tumour cells. Actin and CD31 were not detected in tumour cells but were detected in blood vessels.

### Follow-up

The postoperative clinical evolution was favourable, including remission of the exophthalmia (Fig. 1d) but persistent diplopia and limiting adduction and descent of right eye that improved over time. No evidence of residual tumour was found in the orbital magnetic resonance control (Fig. 1e,f). Fifteen months after surgery, no signs of recurrence were noticed. The patient remains under close follow-up.

## Discussion

SFTs represent a diverse group of ubiquitous rare spindle cell neoplasms with variable clinical behaviour and have not yet been fully explored. SFT was first described in 1931 by Klemperer and Rabin, who reported it as a primary neoplasm of the visceral pleura. Although once considered to originate from mesothelial cells, with the advent of immunohistochemistry, a fibroblastic origin, occasionally with myofibroblastic differentiation, is now accepted [4]. Additionally, a possible cellular origin from a fibroblastic differentiated cell type in mesenchymal tissue that includes endoneural dendritic cells, dermal dendrocytes, or pericytes has been suggested [5]. These tumours occur worldwide, and all races and ethnic groups are affected [6]. In the lacrimal area, SFTs arise from the mesenchymal cells of the periductal connective tissue of the lacrimal gland or from the mesenchymal elements of the lacrimal gland fossa [7]. The previously reported cases suggest that most orbital SFTs occur in middle-aged adults without an obvious association with sex. However, SFTs of the lacrimal gland and lacrimal fossa tend to occur at a younger age, with an average in the third decade, show a male predominance, and commonly have left orbit involvement [8]. Our case does not overlap with this pattern.

### Clinical data

All patients with lacrimal SFTs present one or more symptoms relating to a growing tumoural mass at the site of the lacrimal gland. This may be accompanied by upper eyelid swelling, occasionally limited ocular motility, unilateral eyeball displacement and facial deformity. In our case, there was no palpable mass. Sometimes, localization of the tumour in the lacrimal area causes eyeball protrusion, leading to misinterpretation as endocrine exophthalmia and the referral of the patient to an endocrinology service, as occurred in our case. These tumours have previously tended to grow slowly, form a single mass, and sometimes exert compressive effects on the adjacent viscera or neurovascular structures; the exception was one case that did not exceed 3.5 cm in size.

### Imaging findings

In addition to a complete physical examination, affected patients should undergo imaging tests that may include X-rays, ultrasound, computed tomography scan or magnetic resonance imaging (MRI). In the previously reported cases, a well-demarcated mass with regular margins and no infiltrating adjacent structures or bone remodelling was described. MRI features can be variable, are correlated with cellular components and collagen, degenerative changes, haemorrhage and necrosis within the tumours. Compared with the cerebral cortex or extraocular muscle, SFTs in the lacrimal area show isointense signals on T1-weighted images and variable signals on T2-weighted images [9]. In addition, angiography can be used to demonstrate a hypervascularized tumour resembling an angioma [10]. In our case, the tumoural mass was examined by MRI and showed hypointense signal intensity on T1-weighted images and mixed isointense and hyperintense signal intensity on T2-weighted images with cystic lesions with multiple septae, which have not previously been reported in SFTs of the lacrimal gland.

### Histopathological findings

Because of variable clinical presentations and histologic appearances, the diagnosis of SFTs can be a challenge, as we observed in our case, when the result of an extemporaneous examination is not confirmed by the results obtained in paraffin-embedded tissues and immunohistochemistry. SFTs can be misdiagnosed as other mesenchymal lesions (haemangiopericytomas, giant cell angiofibromas, monophasic synovial sarcomas or fibrous histiocytomas), smooth muscle tumours (leiomyomas and leiomyosarcomas), neural tumours (gliomas, fibrous meningiomas, schwannomas, or neurofibromas), etc. In the past, some SFTs have probably been misdiagnosed as other spindle cell neoplasms. An extensive histopathological analysis of 41 fibroblastic lesions of the orbit that had different diagnoses, including haemangiopericytomas, fibrous histiocytoma, mixed tumours and giant cell angiofibroma, led to their reclassification as SFTs [11].

Histopathological and immunohistochemical staining features and, more recently, molecular analyses are the basic tools used to diagnose SFTs and also have prognostic value. Additionally, fine needle aspiration has been mentioned as useful for diagnosing SFT [12]. Similar to other locations, SFTs occurring in the lacrimal fossa can histologically mimic haemangiopericytoma [13]. The histological differentiation of these two types of lesions is difficult because cases of haemangiopericytoma can show features of SFT and vice versa [14–16]. Many pathologists consider haemangiopericytoma and solitary fibrous tumour to represent a continuum of tumours [17]. Solitary fibrous tumours and haemangiopericytomas overlap, even if they are not identical entities. For this reason, the WHO has created a combined term, SFT/haemangiopericytoma, to describe these lesions. According to the 2016 CNS WHO classification, which uses molecular parameters in addition to histology, the SFT/hemangiopericytoma classification sets three tumour grades: grade I (often diagnosed as SFT), grade II (corresponding typically to haemangiopericytoma) and grade III (previously termed anaplastic haemangiopericytoma) [18]. The differential diagnosis of haemangiopericytoma is mandatory due to its malignant course and its rate of recurrence and metastasis, which contrast with the benign evolution of SFT. In our case, spindle cells with long processes (fibroblast-like cells) and minimal cytoplasm, small elongated nuclei and small visible nucleoli were observed to have a haphazard distribution. The stroma was collagenized, with thick bands of collagen interspersed between the tumour cells. Branching staghorn vasculature, similar to that seen in haemangiopericytoma, was found. Mature adipocytes and giant multinucleated stromal cells, which have been reported in other cases, were absent [19]. The histological criteria for malignancy include tumours greater than 5 cm, cellular pleomorphism, infiltrative growth, necrosis, haemorrhage, nuclear atypia and high mitotic activity (> 4 mitoses/10 hpf) [18]. In our case, no signs of malignancy were identified, there was no nuclear atypia, and mitotic figures were rare.

### Immunohistochemical findings

Immunohistochemical tests are necessary to confirm the diagnosis. CD34 has over time become considered the most sensitive marker for SFTs (95–100%); therefore, to call a tumour an ‘SFT’, it should be CD34-positive [2]. In all cases reported until now, the SFTs of the lacrimal area showed uniform positivity for CD34 and vimentin. Other markers, including Bcl-2 and CD99, were variably expressed. Markers such as desmin, SMA, EMA, CK and S100 protein, were negative. In our case, tumoural cells were positive for CD34, vimentin and CD99, infrequently positive for EMA and negative for SMA and CD31, and the Ki67 index was 8%.

### Molecular analyses

Cytogenetic studies are also needed to improve understanding of the clinical and pathological features of SFTs. Although research is underway, some karyotypic aberrations, including both numerical and structural abnormalities, have been reported [20, 21]. Additionally, several studies show that SFTs are characterized by the overexpression of multiple genes, such as genes encoding LDH1, therapeutic targets (kinases and histone deacetylases retinoic acid receptors), and multiple growth factors (PDGF, EGF, VEGF, IGF2, etc.) [22].

Recent molecular analyses suggest that SFTs are positive for nuclear protein STAT6, which is overexpressed due to a paracentric inversion on chromosome 12q13 and NAB2-STAT6 gene fusion [23, 24]. Today, this marker is considered the molecular hallmark of SFT, and it is mandatory in the immunological panel used for the diagnosis of SFTs [25, 26]. To date, this marker has been investigated in only two cases of SFTs in the lacrimal gland. In our case, nuclear STAT6 showed intense and diffuse nuclear staining, supporting the diagnosis of SFT.

Another gene that is upregulated in SFTs is GRIA2, which encodes a subunit of a class of glutamate receptors thought to mediate increased cell proliferation. In a recent study, this marker, which has not yet been researched in lacrimal gland SFTs, was found to be positive in 89% of SFTs and negative in more than 99% of all other histological mimics occurring in soft tissue [27]. However, it should be noted that molecular testing is expensive and not available in every laboratory.

### Treatment

As in other sites of SFTs, effective treatment of SFT of the lacrimal gland and lacrimal fossa means complete resection of the tumour. Sometimes, the entire lacrimal gland may have to be removed, as occurred in our case. No other treatments (radiotherapy or chemotherapy) have proved to be efficient. According to some authors, preoperative arterial embolization can be considered because of the risk of bleeding during surgical resection of these highly vascularized tumours [28].

### Evolution and prognosis

A correct diagnosis is mandatory for proper therapy and management of patients with SFT. Until now, it has been estimated that most extrapleural tumours, with the exception of those of mediastinal origin, have a benign outcome. Usually, recurrences have been attributed, for the most part, to incomplete resection. A review of 189 cases of CNS SFTs revealed that the rate of recurrence in incomplete resection was 58,1%, whereas the rate was 14% in cases of total excision [29]. In addition, SFTs may undergo malignant transformation when not completely excised. Ten to 15% of SFTs are aggressive even when they appear morphologically benign [30, 31].

It is unclear whether SFTs occurring in the lacrimal gland exhibit recurrences or malignant behaviour. To date, no cases with recurrence or metastasis have been reported, suggesting a possible non-aggressive evolution for lacrimal gland SFTs. However, these results should be interpreted with caution because statistical analyses were performed on only a small number of reported cases. Could the recurrence or malignant behaviour of these tumours be correlated with their morphological features? Although orbital SFTs are mostly solid tumours, partial or complete cystic changes have been reported in seven previous cases (three primary and four recurrent), but none of these occurred in the lacrimal gland. [32–36]. Although the mere presence of cavitary lesions does not necessarily indicate aggressive behaviour, cystic degeneration poses a risk of recurrence and may suggest malignant transformation over time, especially in recurrent tumours. [35]. The mechanism of cystic degeneration is unknown, but it has been suggested that the tumour outgrows its blood supply, leading to necrotic areas that are later reabsorbed, generating cystic spaces [32]. No data are available to establish whether there is a relationship between tumour age and the onset of cystic degeneration.

## Conclusions

With new advances in medical research, more cases of orbital solitary fibrous tumours, including SFTs in the lacrimal area, are being diagnosed and reported. To date, in all previously reported cases, SFTs of the lacrimal gland were solid tumours, and cystic changes were not been described prior to our case. Cystic degeneration of the tumour requires long-term follow-up because recurrence at the site of surgery or metastasis can occur several years after the excision of the primary tumour, even in tumours that behave benignly.

## Data Availability

All data generated or analysed during this study are included in this published article and its supplementary information files.

## Abbreviations

Bcl-2: B-cell leukaemia-2
BMI: Body mass index
CD: Cluster of differentiation
CK: Cytokeratin
EGF: Epidermal growth factor
EMA: Epithelial membrane protein
GRIA2: Glutamate ionotropic receptor AMPA type subunit 2
IGF: Insulin-like growth factor
LDH1: Aldehyde dehydrogenase 1
MRI: Magnetic resonance imaging
NAB2: NGFI-A binding protein 2
PDGF: Platelet-derived growth factor
SFT: Solitary fibrous tumour
SMA: Smooth muscle actin
STAT6: Signal transducer and activator of transcription 6
VEGF: Vascular endothelial growth factor
WHO: World Health Organization
